# The prognostic significance of PD-L1 expression in patients with glioma: A meta-analysis

**DOI:** 10.1038/s41598-017-04023-x

**Published:** 2017-06-26

**Authors:** Song Xue, Ge Song, Jinming Yu

**Affiliations:** 1 School of Medicine and Life Sciences, University of Jinan-Shandong Academy of Medical Sciences, Jinan, Shandong 250022 China; 2 Department of Radiation Oncology, Shandong Cancer Hospital Affiliated to Shandong University, Jinan, Shandong 250117 China

## Abstract

A large number of studies have shown that programmed death-ligand 1 (PD-L1) is abnormally expressed in gliomas. However, the prognostic significance of PD-L1 expression in glioma patients remains unresolved. Accordingly, we conducted a meta-analysis to determine the prognostic role of high PD-L1 in patients with glioma. Electronic databases were searched to identify studies evaluating PD-L1 expression and overall survival (OS) in these patients. A total of 6 studies (published in 4 articles) that involved 1052 patients were included. Pooled results showed that high PD-L1 expression was associated with worse OS in glioma patients (HR = 1.30, 95% CI: 1.02–1.65, *P* = 0.032). Further subgroup analysis indicated that high PD-L1 expression in glioblastoma (GBM) was also associated with worse OS (HR = 1.40, 95% CI: 1.03–1.90, *P* = 0.030). Conversely, in index subgroup analysis, neither PD-L1 protein (HR = 1.43, 95% CI: 0.97–2.10, *P* = 0.068) nor gene (HR = 1.20, 95% CI: 0.83–1.74, *P* = 0.322) expression was significantly associated with OS. PD-L1 may represent a promising biomarker that predicts disease progression in patients with glioma or GBM. However, because of our limited sample size, further prospective or retrospective multi-centre, well-designed studies should be performed to verify this result.

## Introduction

Gliomas are the most common primary central nervous system (CNS) tumour, representing 40% of all brain tumours^1^. According to the World Health Organization (WHO) 2007 classification, gliomas are divided into grades I–IV, with each higher grade corresponding to an increased level of malignancy and poorer overall survival (OS). Glioblastoma (GBM, glioma grade IV) is the most malignant and aggressive form of primary brain tumour. Despite the use of a standard treatment regimen, including total surgical resection, radiotherapy, and adjuvant chemotherapy, GBM remains a major clinical challenge, with an overall 5-year survival rate of only 9.8%^2^. One significant challenge to treating GBM is the inevitable recurrence of these tumours. It is therefore very important to obtain an accurate prognosis while the tumour is in an early stage.

Programed death-ligand 1 (PD-L1) is a T cell costimulatory molecule implicated in tumour immune escape mechanisms. Binding of PD-L1 to its receptor (PD-1) induces apoptosis or exhaustion of activated immune cells^3^. Observed in human malignancies, aberrant expression of PD-L1 is associated with clinicopathological features in several types of cancer, including colorectal^4^, breast^5^, and lung cancers^6^. Blocking PD-L1 or PD-1 has been demonstrated to be among the most promising immunotherapeutic strategies for boosting the immune system’s ability to combat cancer^7, 8^. Although clinical anti-PD-L1/PD-1 therapies have been developed, data regarding the prognostic role of PD-L1 expression in glioma are limited, and findings to date are controversial. Several studies have found that high PD-L1 expression on glioma cells correlates with poor prognosis^9, 10^, though not all reports support this conclusion^11, 12^. Thus, it would be useful to determine whether PD-L1 expression is associated with the prognosis of glioma patients.

In this study, we conducted a meta-analysis to evaluate the prognostic role of PD-L1 in patients with glioma. To our knowledge, this is the first meta-analysis to quantitatively synthesize information from all previously published studies related to the potential prognostic value of PD-L1 in glioma patients.

## Methods

### Literature Search Strategy

All relevant articles were retrieved from searches of the PubMed, EMBASE, Ovid and Cochrane Library databases. These searches were performed using the following keywords and text words: (“PD-L1”, “Programmed cell death ligand 1”, “B7-H1”) and (“glioma” or “glioblastoma” or “central nervous cancer” or “CNS cancer”). The final search in this study was updated on October 15, 2016. References in eligible studies or textbooks were further manually reviewed to identify additional potentially eligible studies.

### Inclusion and Exclusion Criteria

Published studies were selected for analysis using the following inclusion criteria: (1) the study patients were confirmed by the department of pathology using diagnostic criteria for glioma, and gliomas were classified based on current WHO guidelines; (2) the study explored the prognostic significance of PD-L1; (3) the article was published in English. The exclusion criteria included the following: (1) duplicate studies; (2) letters, comments, meeting abstracts and reviews; (3) laboratory studies or animal studies or studies related to other types of CNS cancer.

### Data Extraction

The following data from each enrolled study were extracted: the first author’s name, year of publication, country of origin, tests used for indexing, technique used to assay PD-L1 levels, cut-off value used to determine PD-L1 positivity and survival analysis method. Hazard ratios (HRs) for PD-L1 expression and OS and the corresponding 95% confidence intervals (95% CIs) were extracted from tables or Kaplan-Meier curves for both PD-L1 low/negative (control group) and PD-L1 high/positive (experimental group) expressers. When both univariate and multivariate analyses of OS results were performed, HRs and 95% CIs were extracted preferentially from the multivariate analysis. When HRs were not reported, we used a previously published methodology^13^ in Engauge Digitizer version 4.1 to estimate the logarithm-transformed HR and determined variance based on Kaplan–Meier curves.

### Quality Assessment of Studies Included

Two reviewers (Song Xue and Ge Song) independently assessed the quality of the selected papers using a modified Newcastle-Ottawa Scale. The scale consisted of three items describing the subject selection criteria (0–4 points), comparability of subjects (0–2 points), and outcomes (0–3 points). The full score attainable was 9 points. In addition, articles with a total score of more than 5 points were considered to be of high quality^14^.

### Statistical Analysis

HRs and 95% CIs were used to measure the effect size. An HR greater than 1 indicated a poor prognosis for patients with high/positive PD-L1 expression. The impact of PD-L1 on survival was considered to be statistically significant if the 95% CI for the HR did not overlap 1. Heterogeneity among studies was evaluated using Cochrane’s Q tests (Chi-squared tests) and the I^2^ metric. I^2^ > 50% or *P* < 0.10 in a Q test indicated large heterogeneity between studies, and we used random effects models to calculate the pooled HR and 95% CI. The fixed effects model was utilized for the remaining analyses. Subgroup analyses were applied to identify the sources of any observed heterogeneity. Such analyses were conducted for the GBM patients, index for PD-L1 protein and gene expression, cut-off value to indicate tumour PD-L1 positivity, and survival analysis method. All analyses were performed using Stata Version 12.0 (Stata Corporation) and Cochrane Review Manager version 5.3 (Cochrane Collaboration). All statistical tests were two sided, and *P* < 0.05 was taken to indicate statistical significance.

## Results

### Search Results

**Figure 1:**
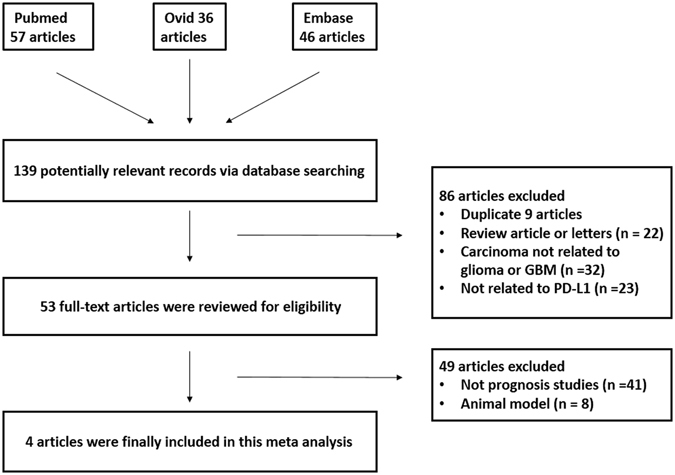
Flow diagram of the literature search and study selection protocols.

Using a previously established retrieval strategy, 139 potentially relevant articles were initially identified from the databases searched. Eighty-six manuscripts were then excluded from the analysis for the following reasons: 9 were duplicate, 22 were reviews or letters, 32 concerned a carcinoma not related to glioma or GBM, and 23 were not related to PD-L1. Thus, 53 articles remained for further full-text assessment. After careful reading of the full text, 49 of the reports were excluded because they did not provide a prognosis (n = 41) or were based on an animal model (n = 8). Ultimately, 4 articles were included in this meta-analysis (Fig. 1)^9–12^.

### Characteristics of the Included Studies and Quality Assessment

Two of the selected articles each have enrolled two cohorts, and two articles each were selected to assess the prognostic value of expression of the PD-L1 protein and PD-L1 gene^10, 11^. There was no overlap between the cohorts enrolled in each article. Thus, 6 studies (from 4 articles) involving 1052 patients were available for this meta-analysis. All of the relevant features in the 6 eligible studies are listed in Table 1. The publication years of the eligible studies ranged from 2013 to 2016. Five of the studies evaluated GBM (WHO IV), and the remaining study evaluated glioma (WHO I–IV). Two of the studies evaluated PD-L1 at the gene level, and four evaluated it at the protein level. In one of the gene-level studies, the researchers applied Agilent microarray techniques to assess PD-L1 gene expression, whereas Illumina RNASeq was used in the other. For the protein-level studies, PD-L1 expression was detected using immunohistochemistry (IHC) in 3 and immunofluorescence histochemistry in the other. Three studies reported the observed staining pattern. PD-L1 expression was detected either on the cell surface or in the cytoplasm. The cut-off criterion used to evaluate positivity for PD-L1 protein expression was the percentage of cells stained (≥5%) in 3 studies and the simple presence of PD-L1 staining in 1 study. Among the included protein studies, the percentage of stained cells (≥5%) was therefore the most common cut-off value used to evaluate positive PD-L1 expression. Only two studies conducted multivariate analysis of OS, and remaining four conducted univariate analysis. The rates of patients with gliomas that were positive for PD-L1 expression ranged from 35.29% to 61. 70%.

**Table 1:** Main characteristics of four studies included in meta-analysis.

First author (y)	Country	Index	Patients	Material	Assay	Staining pattern	Cut off^§^	Number	PD-L1 (+/−) NO.	Positive (%)	Analysis method	HR^#^ (95% CI)	NOS scale
Liu, Y.^9^	Denmark	Protein	Glioblastoma	NM	IFC	NA	Non-percentage^¶^	17	6/11	35.29	UA	4.67 (1.42–15.43)	6
Berghoff, A. S.^11^^†^	Austria	Protein	Glioblastoma	FFPE	IHC	Membranous	Percentage (≥5%)	117	44/73	37.61	UA	1.22 (0.83–1.8)	7
		Gene	Glioblastoma	TCGA	Agilent microarray	—	Median PD-L1 expression levels	446	223/223	50	MA	1.036 (0.87–1.23)	
Nduom, E. K.^15^^†^	USA	Protein	Glioblastoma	TMA	IHC	Membranes	Percentage (≥5%)	94	58/36	61.70	UA	1.74 (1.09–2.77)	7
		Gene	Glioblastoma	TCGA	Illumina RNASeq	—	0.37	149	NM	NM	MA	1.52 (1.03–2.25)	
Zeng, J.^12^	China	Protein	Glioma	TMA	IHC	Membranes/Cytoplasm	Percentage (≥5%)	229	117/112	51.09	UA	1.05 (0.76–1.47)	8

Note: ^†^This research contains two cohorts, one is protein cohort, and the other is gene cohort.

^§^Cut off criterion for PD-L1 Positive.

^¶^>10 cells/field tumor cells.

^#^HR for OS and positive PD-L1 expression.

Abbreviations: IHC, Immunohistochemistry; IFC, Immunofluorescence histochemistry; FFPE, Formalin-fixed paraffin-embedded; TMA, tissue microarray; TCGA, The Cancer Genome Atlas.

UA, univariate analysis; MA, multivariate analysis; HR, Hazard ratio; CI, Confidence interval; NM, Not mentioned; NOS, Newcastle-Ottawa scores.

The quality of the selected studies was assessed by using the Newcastle-Ottawa Scale and found to range from 6 to 8, indicating that the studies were of high quality (Table 2).

**Table 2:** Quality assessment of eligible studies using the Newcastle-Ottawa quality assessment.

Lead author (y)	Selection^†^	Comparability^‡^	Outcome^§^	Total scores^¶^
Liu, Y. *et al*.^9^	3	1	2	6
Berghoff, A. S. *et al*.^11^	3	2	2	7
Nduom, E. K *et al*.^10^	3	2	2	7
Zeng, J. *et al*.^12^	3	2	3	8

^†^Selection (0~4 points).

(1) Representativeness of the exposed cohort (1 point, truly or somewhat representative of the average level in the community; 0 point, selected group of users or no description of the derivation of the cohort).

(2) Selection of the non-exposed cohort (1 point, drawn from the same community as the exposed cohort; 0 point, drawn from a different source or no description of the derivation of the non-exposed cohort).

(3) Ascertainment of exposure (1 point, secure record or structured interview; 0 point, written self-report or no description).

(4) Demonstration that outcome of interest was not present at start of study (1 point, yes; 0 point, no).

^‡^Comparability (0~2 points) (2 points, study controls for the most important factor and any additional factor; 1 point, study controls for the most important factor or any additional factor; 0 point, study controls without the most important factor or any additional factor).

^§^Outcome (0~3 points).

(1) Assessment of outcome (1 point, independent blind assessment or record linkage; 0 point, self-report or no description).

(2) Was follow-up long enough for outcomes to occur (1 point, yes; 0 point, no).

(3) Adequacy of follow up of cohorts (1 point, complete follow up or subjects lost to follow up unlikely to introduce bias; 0 point, follow up rate <80% and no description of those lost, or no statement).

^¶^The quality score was ranked as low (≤5points) or high (≥6 points).

### Correlation between OS and PD-L1 expression in glioma and GBM

**Figure 2:**
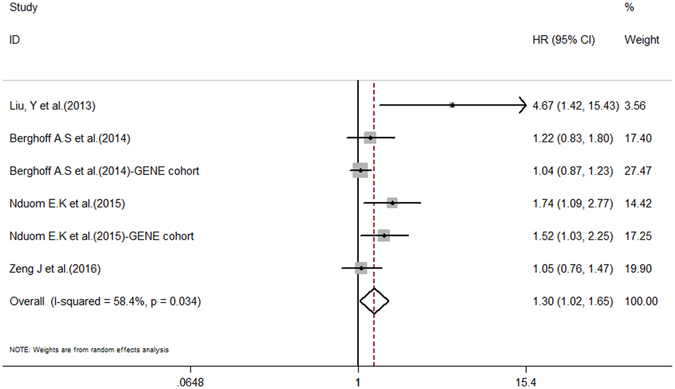
Forest plot illustrating the relationship between PD-L1 expression and OS in glioma patients. Each study is accompanied by a point estimate of its hazard ratio and 95% confidence interval (extending lines). The diamonds represent the estimated pooled effect (labelled ‘total’).

**Figure 3:**
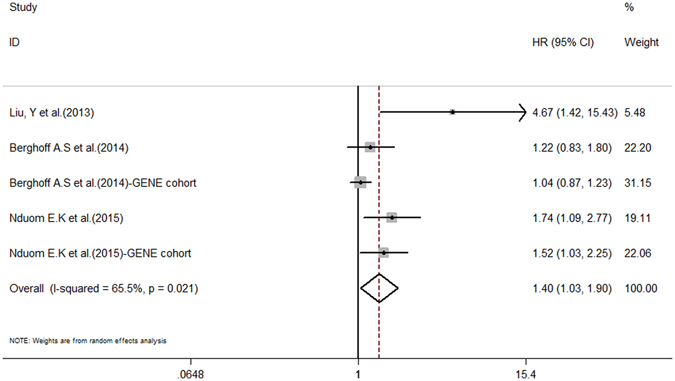
Forest plot for relationships between PD-L1 expression and OS in glioblastoma patients.

The combined HR for the six studies evaluating PD-L1 expression and it relationship with OS was 1.30 (95% CI: 1.02–1.65, *P* = 0.032), suggesting that high/positive PD-L1 expression is an indicator of a poor prognosis in glioma patients. Fig. 2 shows a forest plot of the association between PD-L1 and OS in glioma patients. As significant heterogeneity among the studies was observed (Cochran’s Q, *P* = 0.034, I^2^ = 58.4%), subgroup analysis of GBM patients was performed. A total of five studies were included in the GBM patient sub-group analysis (Table 3), which was performed using a random-effects model (*P* = 0.021, I^2^ = 65.5%). Our results revealed an HR for OS and positive PD-L1 expression of 1.40 (95% CI: 1.03–1.90, *P* = 0.030) (Fig. 3).

**Table 3:** A summary of hazard ratios (HRs) for subgroup analyses of PD-L1 expression of glioma patients.

Subgroup analysis	No. of studies	No. of patients	Model	Pooled HR (95% CI)	*p* value	Heterogeneity (I^2^)	*P* value	Subgroup diffrence P value
Patients								—
GBM	5	823	Random	1.40 (1.03–1.90)	0.030	65.5%	0.021	
Index								0.530
Protien	4	457	Random	1.43 (0.97–2.10)	0.068	60.7%	0.054	
Gene	2	595	Random	1.20 (0.83–1.74)	0.322	67.7%	0.079	
Cut off								—
Percentage (≥5%)	3	440	Fixed	1.24 (0.99–1.54)	0.061	33.5%	0.222	
Analytical method								0.530
Univariate	4	457	Random	1.43 (0.97–2.10)	0.068	60.7%	0.054	
Multivariate	2	595	Random	1.20 (0.83–1.74)	0.322	67.7%	0.079

### Sub-group analysis

**Figure 4:**
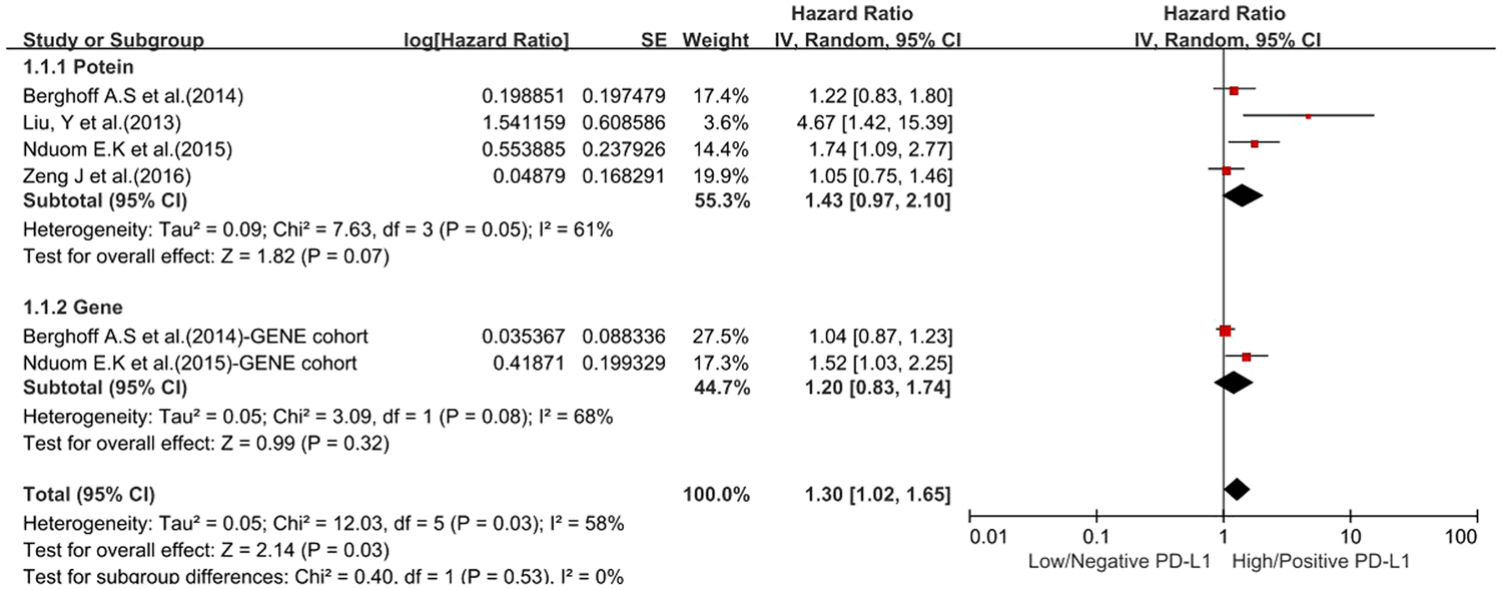
Forest plot for the association between PD-L1 expression and OS in terms of subgroup analysis of the testing index.

**Figure 5:**
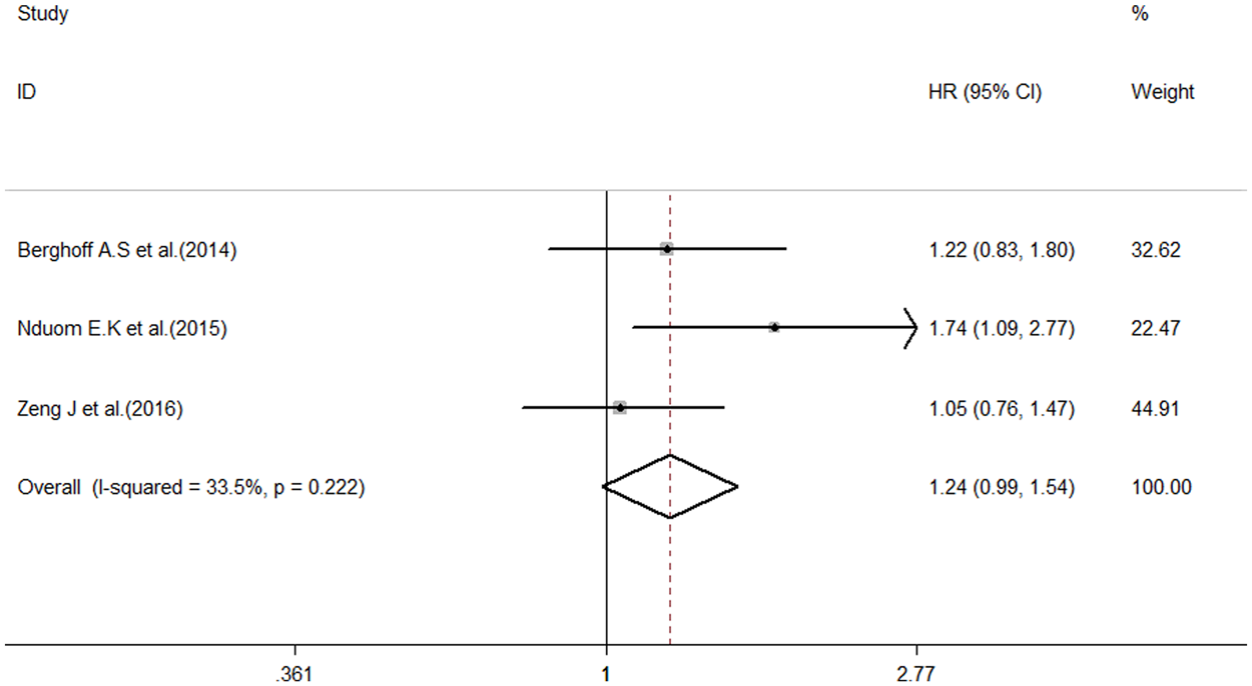
Forest plot for the association between PD-L1 expression and OS in terms of subgroup analysis of the cut-off criterion.

To obtain further insight, we performed subgroup analysis of the testing index, analytical method, and cut-off value to evaluate the prognostic significance of PD-L1 expression in glioma patients. The subgroup analysis of the testing index revealed a merged HR for PD-L1 protein expression of 1.43 (95% CI: 0.97–2.10, *P* = 0.068), whereas that for PD-L1 gene expression was 1.20 (95% CI: 0.83–1.74, *P* = 0.322). There was no statistically significant difference between these two groups (*P* for subgroup difference = 0.530) (Fig. 4). Three of the eligible studies were enrolled in our subgroup analysis of cut-off values (≥5%). High/Positive PD-L1 expression was associated with worse OS (HR = 1.24, 95% CI: 0.99–1.54), but these results were not significant (*P* = 0.061) (Fig. 5), and the studies showed moderate heterogeneity (*P* = 0.222, I^2^ = 33.5%). The results of our subgroup analysis of the analytical method were the same as those of the testing index subgroup analysis (See Supplementary Figure S1). A summary of the hazard ratios obtained in the subgroup analyses is shown in Table 3.

### Sensitivity Analysis

**Figure 6:**
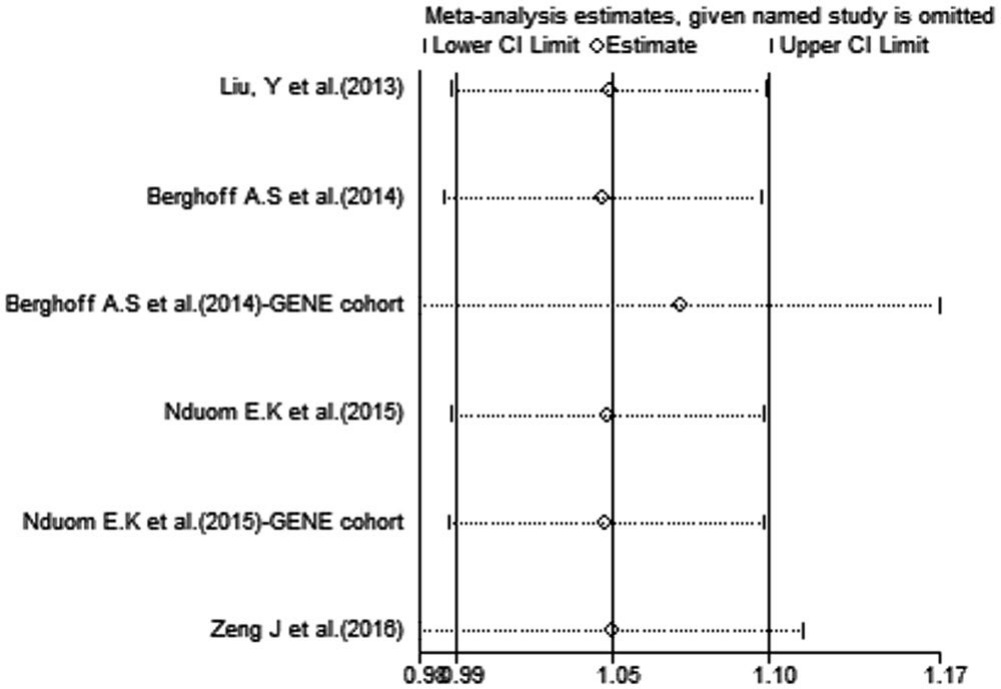
Sensitivity analysis of the relationship between PD-L1 and OS.

The selected studies were sequentially removed to determine whether any single study influenced the pooled results. As shown in Fig. 6, the stable pooled HRs were not significantly affected by any individual study.

## Discussion

Both glioma cell lines and glioma cells in tissue samples have been found to aberrantly express PD-L1^15–17^. Expression of PD-L1 may affect tumour cell oncogenic signalling pathways that have been collectively termed an “innate immune resistance” mechanism. Parsa *et al*. found that glioma cells lines (SF126, SF210, U87, U251, and U373) with genetic deletions or mutations in phosphatase and tensin homologue (PTEN) had higher PD-L1 protein levels than cells wild-type for PTEN^18^. In addition, PD-L1 expression levels in cells in the tumour microenvironment exposed to cytokines were markedly higher, which has been termed an “adaptive immune resistance” mechanism. One example of adaptive immune resistance is an increase in the level of PD-L1 expression in glioma cell lines after the addition of IFN-γ^16^.

Increased PD-L1 levels have been associated with a poor prognosis in several cancers, such as nasopharyngeal carcinoma^19^, gastric carcinoma^20^, and lung cancer^21^. In contrast, there are no consistent results for the prognostic value of PD-L1 in glioma. In a study of 229 glioma patients (grades I–IV), Zeng *et al*. found no significant associations between PD-L1 and prognosis by univariate analysis. When the median survival time (12 months) was used as a cut-off value, they found that positive PD-L1 expression had a significant impact on poor OS in patients who survived or were followed up for 12 months^12^. Liu *et al*. first sought to determine the prognostic value of PD-L1 in tumour masses (TMs) collected from patients with GBM. A higher level of PD-L1 expression (>10 PD-L1+ cells) in a TM was significantly correlated with a poor prognosis, whereas no or low levels of PD-L1 (1–9 PD-L1+ cells) were not^9^. An increasing number of researchers have performed studies aimed at investigating the prognostic value of PD-L1 in GBM, but the results thus far have been inconsistent. For example, using IHC to analyse 135 GBM specimens, Berghoff *et al*. found no significant associations between a membranous staining pattern for PD-L1 expression and OS^11^. The same approach was taken by Nduom *et al*., who investigated the prognostic role of PD-L1 expression in 94 patients with GBM and found that high/positive PD-L1 expression was associated with significantly shorter survival (*P* = 0.0086). Additionally, by applying a multivariate Cox proportional hazards regression model, they found low/negative PD-L1 expression to be a protective factor for OS (HR = 0.554; *P* = 0.018)^10^. Moreover, both of these groups analysed the GBM dataset at The Cancer Genome Atlas (TCGA) and reached contradictory conclusions regarding the prognostic value of PD-L1 gene expression. Berghoff *et al*. used a level 2 Agilent microarray to analyse a cohort of 446 patients, and the results showed no prognostic value of PD-L1 gene expression^11^. In contrast, Nduom *et al*. utilized level 3 Illumina RNASeq and found a significant correlation between high levels of PD-L1 gene expression and unfavourable prognoses. Multivariate analysis demonstrated that PD-L1 independently and negatively impacted survival (HR = 1.52; 95% CI = 1.03–2.25; *P* = 0.0343)^10^. Therefore, meta-analysis of the data available regarding the prognostic significance of PD-L1 would be useful and is urgently needed.

The present meta-analysis is the first to estimate the correlation between PD-L1 overexpression and survival in patients with glioma. In this meta-analysis, we combined the statistics reported in 4 articles (6 studies) including 1052 patients with glioma, and the data indicated that PD-L1 expression plays a significant role in predicting OS in these patients. We found that the combined HR (1.40) for the GBM data alone was larger than that for all 6 eligible studies of glioma (1.30), suggesting that PD-L1 expression could serve as an important prognostic factor for malignant glioma. However, this result should be considered cautiously because heterogeneity was not eliminated in our GBM subgroup analysis of OS.

Based on subgroup analysis of the testing index, positive PD-L1 expression, at both the protein and gene levels, was associated with poor OS, but this relationship was not significant (*P* = 0.068 and *P* = 0.322, respectively). In addition, a strong tendency toward statistical significance was observed between positive PD-L1 expression and a worse prognosis in the cut-off subgroup analysis (HR = 1.24, 95% CI 0.99–1.54, *P* = 0.061), suggesting that a cut-off value of 5% can be used to estimate OS. Nevertheless, several concerns should be addressed regarding these results. We could not eliminate heterogeneity in the testing index subgroup analysis of OS. There is a lack of validated assays for quantifying the level of PD-L1 expression, and no standardized cut-off criteria are currently applied when determining whether PD-L1 expression is positive. These factors may underlie the heterogeneity observed among the included studies^22^. In addition, the results of the subgroup analysis of cut-off values should be interpreted with caution because a relatively small number of studies (3 studies) used 5% as the cut-off value. Hence, additional analyses are needed to develop better sensitivity, and specific assays are needed to detect PD-L1 expression^23^.

Several important limitations should be considered when interpreting our analysis. First, we only included articles published in English. Additionally, we did not analyse publication bias because the number of included studies was relatively small (<10); this likely introduced bias. Moreover, we could not extract HRs with 95% CIs from some of the studies; thus, we evaluated HRs using Kaplan-Meier curves. Finally, we were unable to evaluate the prognostic significance of PD-L1 by stratifying patients according to their clinical features because most of the primary studies did not provide sufficient information to analyse correlations between PD-L1 expression and clinical features, such as age, sex, Karnofsky Performance Status, and O-6-methylguanine-DNA methyltransferase status. Based on these observations, we believe that additional studies are needed and that our conclusions should be interpreted with caution.

In conclusion, despite the limitations described above, our meta-analysis is the first report to focus on the prognostic significance of PD-L1 expression in glioma patients. Our study demonstrated high/positive PD-L1 expression to be associated with poor OS in patients with glioma, and this effect remained significant when the analysis was restricted to GBM patients. Furthermore, our sensitivity analysis did not indicate that alterations in the results (HR) were caused by any individual study, which strengthens the value of our findings. PD-L1 is therefore likely to be particularly useful as a prognostic marker, and it should be included in future validation studies.

## Electronic supplementary material

Supplementary Figure S1
