# Supplementary material for: The prognostic significance of PD-L1 expression in patients with glioma: A meta-analysis
Source: Sci Rep. 2017 Jun 26;7:4231. doi: 10.1038/s41598-017-04023-x (PMC5484664; doi:10.1038/s41598-017-04023-x)
Supplement: Supplementary file 1 — Supplementary Figure S1 [file 41598_2017_4023_MOESM1_ESM.pdf]

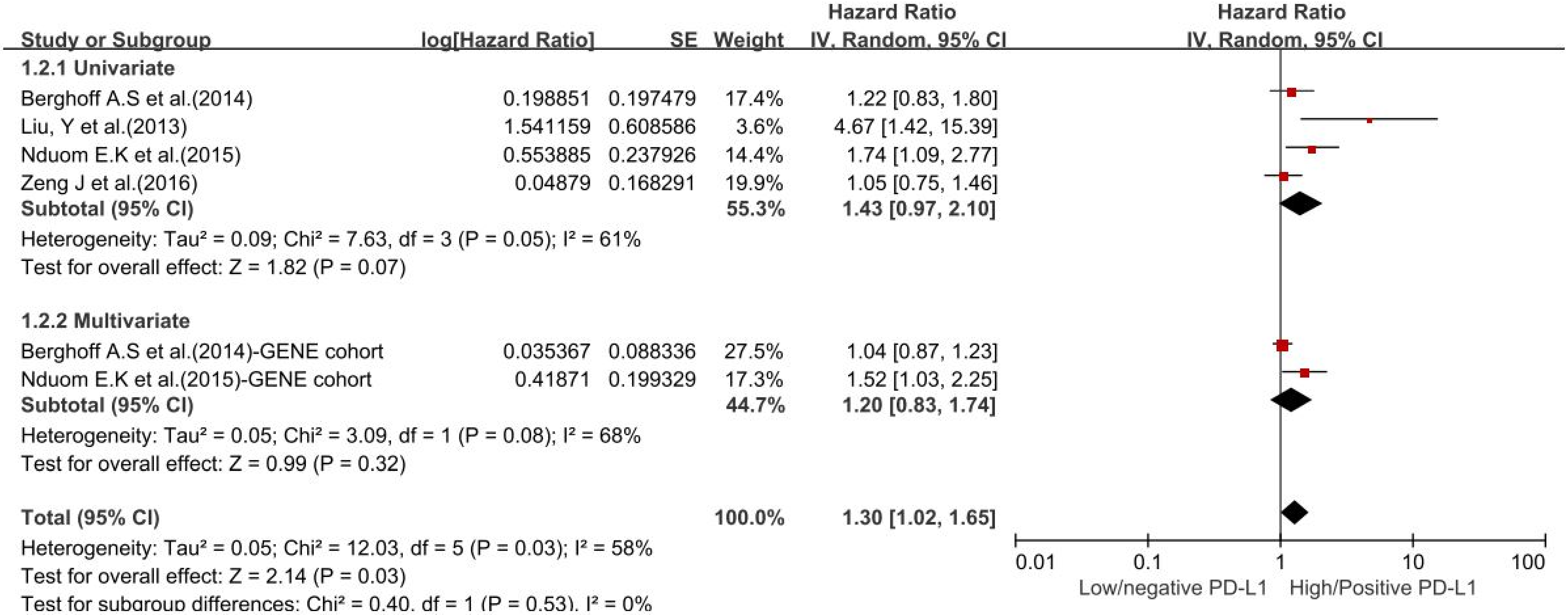

Supplementary Figure S1. Forest plot for the association between PD-L1 expression and OS in terms of subgroup analysis of the survival analysis method.
